# Prognostic Factors and Resectability Predictors in Insular Gliomas: A Systematic Review

**DOI:** 10.1055/s-0043-1769128

**Published:** 2023-08-24

**Authors:** Ariadni Papadopoulou, Niraj S. Kumar

**Affiliations:** 1 Division of Medicine, University College London Medical School, London, United Kingdom of Great Britain and Northern Ireland

**Keywords:** insula, gliomas, prognosis, resectability predictors

## Abstract

**Background** Insular gliomas (INGs) remain a surgically intimidating glioma subgroup encased by eloquent cortical parcels and white matter language tracts, and traversed by multiple middle cerebral artery branches. The predictive power of prognostic factors affecting overall survival (OS), progression-free survival (PFS), and resectability of INGs remain disputed. This comprehensive systematic review analyses prognostic factors and resectability predictors of INGs substantiating pragmatic management options.

**Materials and Methods** A systematic review was conducted in accordance with the Preferred Reporting Items for Systematic Reviews and Meta-Analyses Protocols (PRISMA-P) and the Cochrane Handbook of Systematic Reviews of Interventions guidelines. The PubMed, MEDLINE, and Embase databases were searched in April 2022. All clinical studies with ≥10 patients harboring INGs with any intervention and reporting predictors of OS, PFS, and tumor resectability in INGs were included. Molecular ING prognosticators were also included. Studies combining insular and other gliomas analysis, case studies, experimental and animal studies, conference abstracts, letters to the editor, and articles in other languages were excluded.

**Results** Of the 2,384 articles returned, 27 fulfilled the inclusion criteria totaling 1,985 patients. The review yielded 18 OS and 17 PFS prognosticators. These were classified as preoperative (radiologic; clinical), intraoperative, and postoperative (molecular; histopathologic; clinical) prognosticators. In addition, 21 resectability predictors were categorized as preoperative (radiologic; clinical), intraoperative (surgical approach and assistive technology), and postoperative (histopathologic; clinical). The quality assessment revealed 24/27 studies had low risk of bias. One study with moderate and two studies with high risk of bias were included.

**Conclusion** Negative prognosticators reported in ≥2 studies included putaminal or paralimbic involvement and higher tumor grade, while seizures at presentation, isocitrate dehydrogenase (IDH) mutation, increased extent of resection, and higher Karnofsky Performance Status preoperatively and at 3 months postoperation were positive prognosticators. Resectability predictors reported in ≥2 studies included the positive predictors of zone I/zone IV tumor location and intraoperative imaging use and the negative predictor of encased lenticulostriate arteries. Paralimbic INGs are not a single entity with homogeneous prognosis. Integration of identified prognosticators in a prospective trial to devise a grading system for INGs can improve clinical decision-making.

## Introduction

Despite progress in the management of insular gliomas (INGs) over the past two decades, they remain a disproportionally common and challenging entity, accounting for 25% of all low-grade gliomas (LGGs) 1 with a volume of only 17.4 cm ^3^ . 2 Preferential insular localization of gliomas is hypothesized to stem from a unique microenvironment including developmental, neurochemical, metabolic, and functional features, with distinctive agranular-to-granular anteroposterior transitional cytoarchitecture. 1 While earlier studies associated insular location predominantly with LGGs, recent reports suggest up to 40% of ING lesions are high-grade gliomas (HGGs). 3 Additionally, contradictory survival outcomes have been reported, with earlier studies describing insular lesions as indolent, 3 4 while Singh et al 5 noted shorter median overall survival (OS) in insular glioblastoma compared with superficial tumors. The latter would be consistent with a higher frequency of molecular phenotypes with dismal prognosis such as absence of isocitrate dehydrogenase (IDH) mutations, p53 expression, and *1p19q* codeletion. 6 7 8 9 10 11

Recent literature supports maximum resection as initial management of LGGs. 12 13 However, in subtotal resection (STR) of INGs due to eloquence of the region and efforts to avoid motor and language postoperative deficits may decrease survival advantage. 5 14 It is unsurprising that ING management is challenging and varied across institutions, with some clinicians preferring active surveillance over surgery in LGGs. 15 INGs encompass a distinct subset of clinical entities, where insula-specific prognostic factors and resectability predictors can guide clinical decision-making toward effective and personalized patient care.

Few reviews attempted to address the complexity of ING management, including one by Kim et al reporting age, histology, Yaşargil type 5 with frontal extension, and high extent of resection (EOR) as significant prognostic factors for OS and progression-free survival (PFS). 16 Similarly, a literature review by Hervey-Jumper and Berger identified EOR > 90% as a positive prognosticator and zone I Berger–Sanai tumor location as a positive resectability predictor. 17

The aims of this study include identification of prognosticators to assist in decision-making for patients harboring INGs, and quantifiable operability features assisting maximum safe resection.

## Material and Methods

This systematic review was conducted in accordance with the Preferred Reporting Items for Systematic Reviews and Meta-Analyses Protocols (PRISMA-P) guidelines and the Cochrane Handbook of Systematic Reviews of Interventions. 18 19 20

### Eligibility Criteria

#### Study Types

All clinical studies with ≥10 patients harboring INGs with any intervention and reporting predictors of OS, PFS, and tumor resectability in INGs were included. Molecular ING prognosticators were also included. Studies combining insular and other gliomas analysis, case studies, experimental and animal studies, conference abstracts, letters to the editor, and articles in other languages were excluded.

#### Participants

Studies with patients harboring WHO grade I to IV gliomas, 21 including astrocytoma, oligodendroglioma, or glioblastoma phenotypes involving the insular cortex were included.

#### Interventions

Studies of therapeutic interventions including chemotherapy, radiotherapy, biopsy, resection, or conservative management with serial neuroimaging were included.

#### Outcomes

Predictors of OS, PFS, and tumor resectability in INGs were the outcomes of interest. Despite not being ING-specific, molecular and histologic prognosticators identified in ING patients were included due to their crucial effect on ING disease progression. OS was defined as the period from initial surgery to death. 22 23 24 25 26 27 PFS was defined as the period from initial surgery to radiologically or clinically defined progression or tumor recurrence. 22 23 24 25 27 Radiologic tumor progression was defined as tumor recurrence, new/increased enhancement on follow-up imaging, increased tumoral volume, midline shift, or mass effect. 28 Clinical progression was defined as new or deteriorating clinical deficit, with symptoms or signs of increased intracranial pressure and cognitive decline. 28

### Literature Search

PubMed, MEDLINE, and Embase databases were searched, with the search covering a period from the inception of the database to April 2022. The detailed search strategy is provided in Supplement A.

#### Screening Process

Titles and abstracts of potentially eligible studies were screened independently by two researchers based on the predetermined inclusion criteria. Subsequently, full text articles were reviewed. Studies were deduplicated using Mendeley 1.19.4.

#### Data Extraction and Synthesis

Data systematically recorded and tabulated included the following: first author; year of publication, title and study design; number of patients; prognostic factor subcategory; effect of a prognostic factor on OS or PFS; resectability; and statistical data ( *p* value, hazard ratio [HR], confidence intervals [CIs], or other). The effect of a prognostic factor was recorded as positive (associated with increased OS/PFS) or negative (associated with decreased OS/PFS). Data were entered on Excel spreadsheets (version 16.29, Microsoft, Redmond, Washington). 29

#### Quality Assessment

Quality assessment was conducted using the Quality in Prognostic Studies (QUIPS) tool. 30 31 32 Items evaluated included the following: study participation, study attrition, prognostic factor measurement, outcome measurement, study confounding, statistical analysis, and reporting. Two independent reviewers assessed the quality of studies. Consensus was reached for all studies.

## Results

**Fig. 1:**
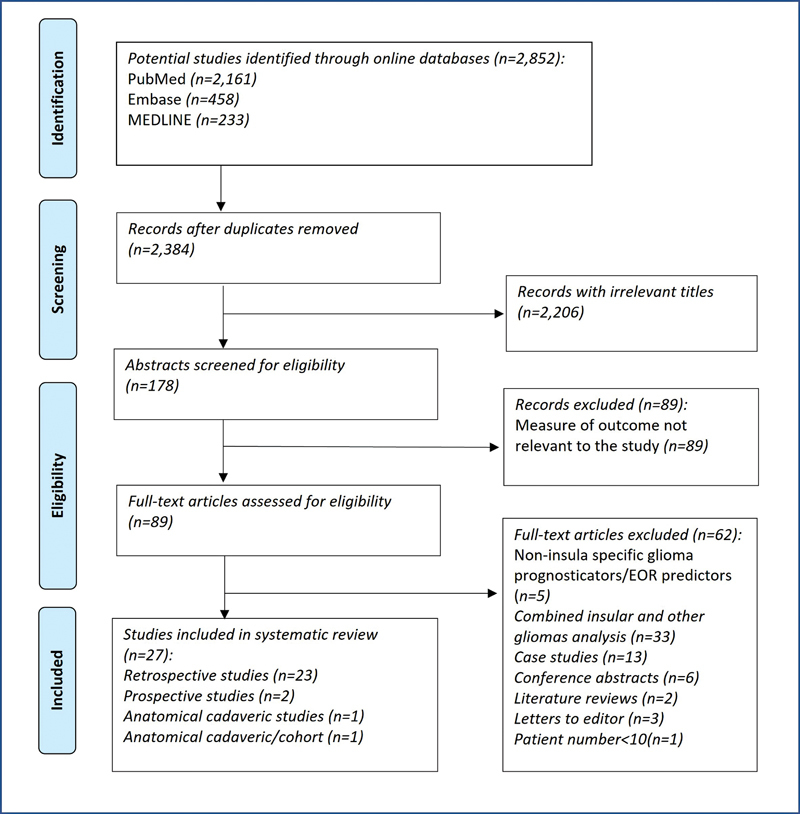
Preferred Reporting Items for Systematic Reviews and Meta-Analyses (PRISMA) flow diagram.

The search identified a total of 2,852 articles. After excluding duplicates ( *n* = 468) and screening titles and abstracts ( *n* = 2,384), 178 potentially eligible studies were found. Eighty-nine full-text articles were extracted, 27 of which met the inclusion criteria ( Fig. 1 ). These included articles studied a total of 1,985 patients.

### Quality Assessment

Twenty-four studies were rated as low risk of bias (RoB), one study was rated as moderate RoB, and two studies were classified as high RoB. The causes of high or moderate RoB were insufficient adjustment for cofounders and inadequate statistical analyses. Quality appraisal results are described in detail in Supplementary Table S1.

### I. Overall Survival and Progression-Free Survival Prognosticators

**Fig. 2:**
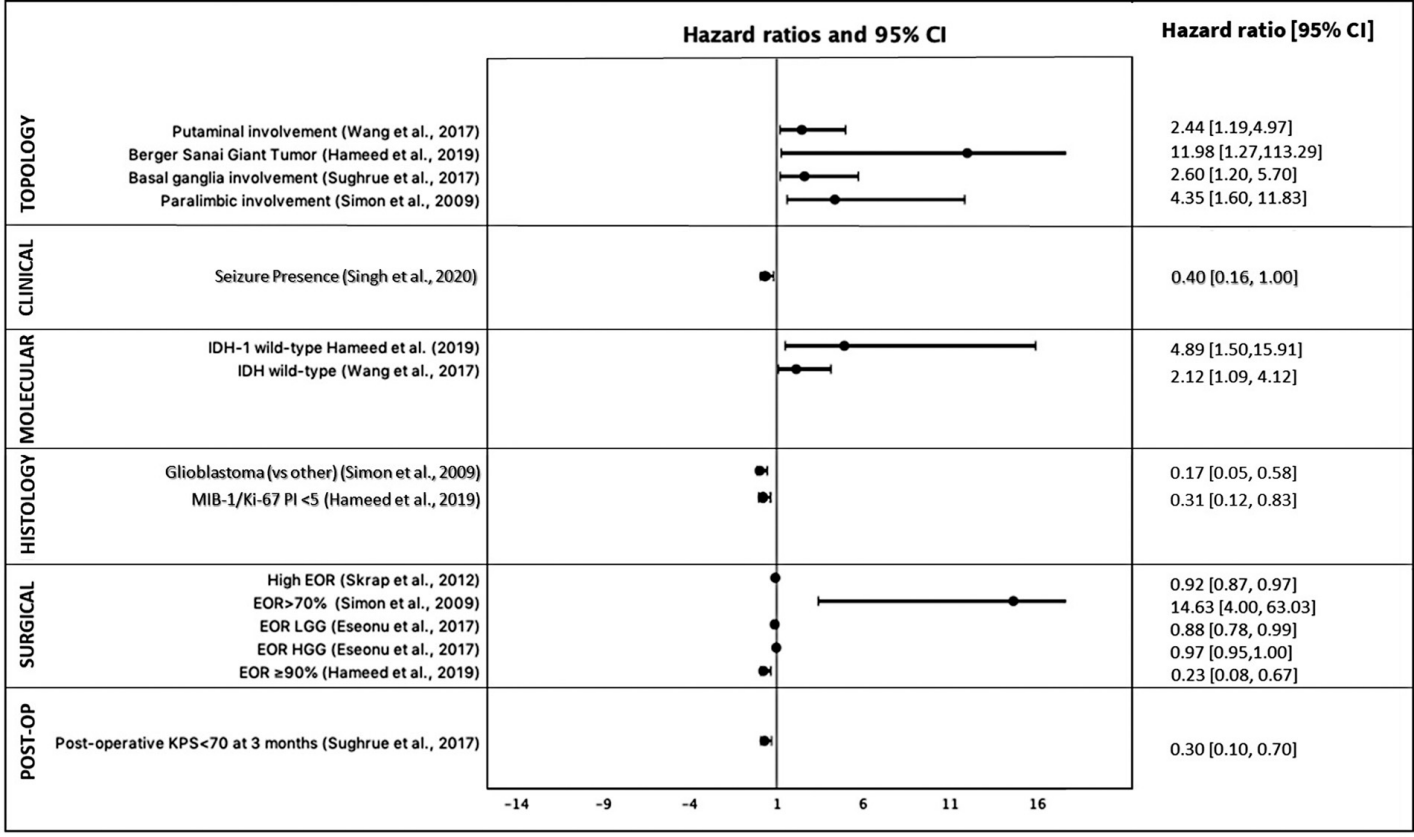
Hazard ratios for overall survival (OS) prognosticators in insular glioma (ING) patients from all reporting included studies. (The figure was created using R Core Team (2020). R: A language and environment for statistical computing. R Foundation for Statistical Computing, Vienna, Austria.)

Of the 27 studies that met the inclusion criteria, 12 studies reported the OS data 5 22 23 24 25 26 27 33 34 35 36 37 and 7 reported the PFS data. 22 23 24 25 27 33 38 A total of 18 OS prognosticators and 17 PFS prognosticators were identified ( Table 1 ). The HRs and CIs of the included OS studies are shown in Fig. 2 .

**Table 1:** Summary of OS and PFS prognosticators of INGs detected in the included studies

Prognosticator		Effect on		Study characteristics		
		OS	PFS	Study	Study design	Patients ( *n* )
**Preoperative**						
**1. Radiologic**						
Putaminal involvement		Negative ( *p* = 0.014)	Negative ( *p* = 0.003)	Wang et al 23	Retrospective	211
Basal ganglia involvement		Negative ( *p* = 0.023)	NR	Sughrue et al 37	Retrospective	72
Berger–Sanai giant tumor		Negative ( *p* = 0.030)	Negative ( *p* < 0.001)	Hameed et al 27	Retrospective	255
Paralimbic involvement		NR	Negative ( *p* = 0.088)	Goze et al 38	Prospective	83
		Negative ( *p* = 0.028)	NR	Tang et al 26	Retrospective	42
		Positive ( *p* = 0.004)	Positive ( *p* = 0.016)	Simon et al 33	Retrospective	95
Nonencased LSAs Not encased		NR	Positive ( *p* = 0.026)	Kawaguchi et al 22	Retrospective	83
**2. Clinical**						
Epilepsy	Seizure presentation (vs. other)	Positive ( *p* < 0.001) a	Positive ( *p* < 0.001) a	Simon et al 33	Retrospective	95
	Preoperative history of seizures	Positive ( *p* = 0.04)	NR	Wang et al 23	Retrospective	211
		Positive ( *p* = 0.048)	NR	Singh et al 5	Retrospective	27
Pre-op KPS score	≥90	Positive ( *p* = 0.02) a	Positive ( *p* = 0.007) a	Wang et al 23	Retrospective	211
	≥90 (vs. 70–80	Positive ( *p* = 0.002)	NR	Schatz et al 36	Retrospective	67
	80–100 (vs. <70)	Positive ( *p* < 0.001)	Positive( *p* = 0.021)	Simon et al 33	Retrospective	95
**Intraoperative**						
EOR	≥90%	Positive ( *p* = 0.019) a	NR	Tang et al 26	Retrospective	42
		Positive ( *p* = 0.009)	NR	Hameed et al 27	Retrospective	255
	>70%	Positive ( *p* < 0.001)	Positive ( *p* = 0.006)	Simon et al 33	Retrospective	95
	High	Positive ( *p* = 0.002)	NR	Skrap et al 34	Retrospective	66
		Positive (LGG: *p* = 0.017)	Positive (LGG: *p* = 0.039)	Eseonu et al 24	Retrospective	74
		Positive (HGG: *p* = 0.020)	Positive (HGG: *p* = 0.024)	Eseonu et al 24	Retrospective	74
	Total resection (vs. partial/biopsy)	NR	Positive ( *p* = 0.008) a	Compes et al 25	Retrospective	43
**Postoperative**						
**1. Molecular**						
IDH–wild-type (vs. mutant)		Negative ( *p* = 0.036)	NR	Tang et al 26	Retrospective	42
		Negative ( *p* = 0.026)	Negative ( *p* = 0.001)	Wang et al 23	Retrospective	211
		Negative ( *p* = 0.008)	NR	Hameed et al 27	Retrospective	255
		NR	Negative ( *p* = 0.009)	Compes et al 25	Retrospective	43
*1p19q* status	Intact	Negative ( *p* = 0.048)	NR	Tang et al 26	Retrospective	42
	Codeleted	NR	Positive ( *p* = 0.014)	Eseonu et al 24	Retrospective	74
*7p* gain and *10q* loss		Negative ( *p* = 0.016)	Negative ( *p* = 0.009)	Compes et al 25	Retrospective	43
Hypermethylated status		NR	Negative ( *p* = 0.009) a	Compes et al 25	Retrospective	43
IDH-wild-type astrocytoma (vs. IDH-mutant *1p/19q* codeleted oligo and IDH-mutant astrocytoma)		NR	Negative ( *p* = 0.009)	Compes et al 25	Retrospective	43
**2. Histopathologic**						
Tumor grade	Higher	Negative ( *p* < 0.001)	NR	Sughrue et al 37	Retrospective	72
	Higher	Negative ( *p* < 0.01)	NR	Capizzano et al 35	Retrospective	50
	WHO III (vs. WHO II)	Negative ( *p* = 0.02)	NR	Kawaguchi et al 22	Retrospective	83
Glioblastoma (vs. other)		Negative ( *p* = 0.004)	Negative ( *p* = 0.017)	Simon et al 33	Retrospective	95
Vimentin positive staining		Negative ( *p* = 0.029)	Negative ( *p* = 0.011)	Compes et al 25	Retrospective	43
MIB-1/K _i_ -67 PI	PI < 5	Positive ( *p* = 0.013)	NR	Hameed et al 27	Retrospective	255
Oligodendroglioma (vs. astrocytoma)		Positive ( *p* < 0.001)	Positive ( *p* < 0.001)	Simon et al 33	Retrospective	95
Neuronal differentiation	Higher	Positive ( *p* < 0.01)	NR	Capizzano et al 35	Retrospective	50
**3. Clinical**						
Permanent (vs. transient) deficit		Negative ( *p* < 0.05)	NR	Hameed et al 27	Retrospective	255
KPS immediately post-op	80–100	Positive ( *p* < 0.001)	Positive ( *p* < 0.001) a	Simon et al 33	Retrospective	95
KPS at 3 mo	80–100	Positive ( *p* < 0.001)	Positive ( *p* < 0.001) a	Simon et al 33	Retrospective	95
	<70	Negative ( *p* < 0.001)	NR	Sughrue et al 37	Retrospective	72

Abbreviations: EOR, extent of resection; HGG, high-grade glioma; IDH, isocitrate dehydrogenase; INGs, insular gliomas; KPS, Karnofsky Performance Status Score; LGG, low-grade glioma; LSAs, lenticulostriate arteries; NR, not reported; OS, overall survival; PFS, progression-free survival; PI, proliferative index.

a Univariate analysis.

### Preoperative Prognosticators

#### Radiologic

The putaminal classification by Wang and colleagues 23 was based on a cohort of 211 participants. Putaminal involvement in magnetic resonance imaging (MRI) was associated with decreased OS (HR = 2.44, *p* = 0.014) and decreased PFS (HR = 2.49, *p* = 0.003). 23 At 2,000 days of follow-up, 83.3% of patients with no putaminal involvement were alive, compared with 50% of patients with putaminal lesions. 23

A study of 72 patients undergoing hyperaggressive resection identified basal ganglia (BG) involvement as a negative OS prognosticator. The 4-year survival of patients with BG involvement was 30% compared with 52% in patients without BG involvement ( *p* = 0.023). 37

**Fig. 3:**
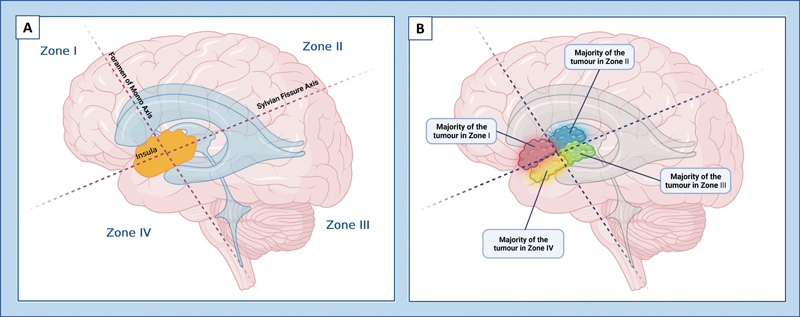
Berger–Sanai classification. ( **A** ) The insula is separated into four zones by an axis corresponding to the sylvian fissure and a perpendicular axis crossing the foramen of Monro. ( **B** ) Tumors are assigned to the zone where >50% of tumor volume is located. Tumors extending to all zones are classified as giant tumors, a negative overall survival (OS), and progression-free survival (PFS) prognosticator. Zone I, IV, and I + IV tumors are positive resectability predictors, while zone II and giant tumors are negative resectability predictors. (The figure was created using BioRender.com ).

Hameed and colleagues 27 investigated the prognostic effect of Berger–Sanai classification ( Fig. 3 ). Giant tumors were a significant negative predictor of OS (HR = 11.98, *p* = 0.030) and PFS ( *p* < 0.001). 27 Patients with ASPI (anterior, superior, posterior, or inferior) zone tumors and giant tumors had an OS of 73.3 (mean) and 60.0 (median) months and a PFS of 57.6 (mean) and 29.0 (median) months, respectively.

**Fig. 4:**
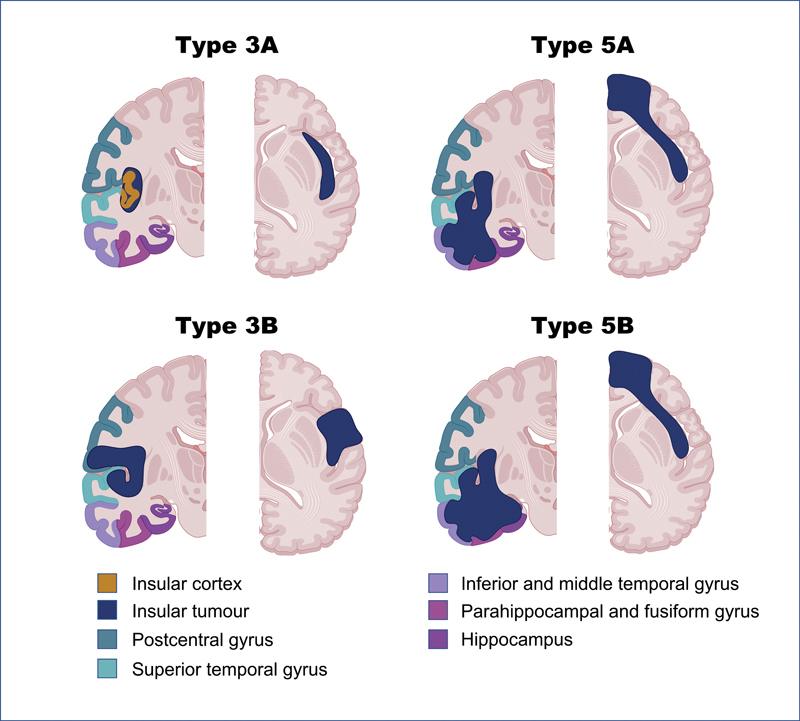
Yaşargil's classification of insular tumors in the coronal (left) and axial (right) views. Type 3A tumors are restricted to the insula, while type 3B tumors also extend to the perisylvian opercula. Type 5A tumors are characterized by paralimbic involvement with frontal and/or temporopolar involvement. Type 5B tumors also have opercular, frontal, and temporopolar involvement with additional hippocampal extension. Please note basal ganglia involvement is possible, although it is not demonstrated in this schematic. (The figure was created using BioRender.com ).

Paralimbic involvement, defined as insular and frontal and/or temporal involvement (Yaşargil types 3B, 5A, and 5B; Fig. 4 ), negatively affected OS in two studies. 26 38 A study by Gozé and colleagues 38 showed paralimbic involvement was associated with decreased PFS with a trend toward significance ( *p* = 0.088). Conversely, Simon and colleagues 33 found paralimbic involvement positively correlated with OS and PFS ( *p* = 0.016). The best prognosis was linked to large frontoinsular (Yaşargil type 5A) and frontoinsulotemporal tumors (Yaşargil type 5A/B) in comparison to other insular tumors for both OS ( *p* = 0.004) and PFS (HR = 3.27, *p* = 0.016).

Kawaguchi and colleagues 22 showed that nonencased lenticulostriate arteries (LSAs) were a positive prognosticator of PFS (OR = 4.3, *p* = 0.026). Postoperative tumor progression observed in patients with nonencased and tumor-encased LSAs was 44.9 and 61.8%, respectively ( *p* = 0.13).

The laterality of the tumor was investigated in three studies but was not a significant prognosticator of OS or PFS. Simon and colleagues 33 reported that location of insular tumors in the dominant or nondominant hemispheres was not associated with a significant difference in OS ( *p* = 0.643) and PFS ( *p* = 0.371). Those findings were corroborated by Wang and colleagues 23 (OS: *p* = 0.059; PFS: *p* = 0.251) and Eseonu and colleagues 24 (OS: *p* = 0.212; PFS: *p* = 0.811).

##### Clinical

Epileptic seizures at presentation were a positive OS 5 23 33 and PFS 33 prognosticator. A cohort study by Wang and colleagues 23 and a study of 27 participants with insular glioblastomas by Singh and colleagues identified preoperative seizure history as a positive OS prognosticator (HR = 0.398, *p* = 0.048). 5 Simon and colleagues 33 reported that presentation with one or multiple seizures, compared with presentation with any other symptom, was associated with improved OS ( *p* < 0.001) and PFS ( *p* < 0.001 _(univariate)_ ).

Three studies reported high preoperative Karnofsky Performance Status (KPS) as a positive OS prognosticator 23 33 36 and two as a positive PFS prognosticator. 23 33 Schätz and colleagues 36 demonstrated that preoperative KPS ≥ 90 is a positive OS prognosticator ( *p* = 0.002, HR = 4.09), while KPS of 70 or 80 was associated with a lower 5-year survival rate of 25 versus 68% in patients with KPS of 90. Wang and colleagues 23 found KPS ≥ 90 to be a positive OS ( *p* = 0.02) and PFS ( *p* = 0.007 _(univariate)_ ) prognosticator. Simon and colleagues 33 also found preoperative KPS 80 to 100 (compared with <70) to be a positive OS ( *p* < 0.001) and PFS ( *p* = 0.021 _(univariate)_ ) prognosticator.

### Intraoperative Prognosticators

In accordance with gliomas affecting other brain regions, seven studies demonstrated greater EOR as a significant positive OS prognosticator 16 17 24 26 27 33 34 and three studies reported greater EOR as a positive PFS prognosticator. 24 25 33

A study of 255 LGG patients demonstrated that patients with EOR ≥ 90% and <90% had a survival of 68.51 (mean) and 49.80 (median) months, respectively ( *p* = 0.009). 27 Furthermore, HGG patients undergoing gross total resection (GTR) and STR had a median survival of 22.00 and 11.30 months, respectively. 27 Eseonu and colleagues 24 noted that higher EOR was identified as a positive PFS prognosticator in LGGs (HR = 0.949, *p* = 0.039) and HGGs ( *p* = 0.024). LGG patients with EOR ≥90% and <90% had a 5-year survival of 100 and 80%, respectively. HGG patients with EOR ≥90% and <90% had a 2-year survival rate of 83.7 and 43.8%, respectively. EOR > 70% was reported as a positive prognosticator of PFS (HR = 8.901, *p* = 0.006) in a study by Simon et al. 33

### Postoperative Prognosticators

#### Molecular

IDH mutation status was a significant prognosticator of OS in four studies 23 25 26 27 and of PFS in two studies. 23 25 Hameed and colleagues 27 identified IDH wild type (IDHwt) as a significant negative OS prognosticator in LGG giant tumors (HR = 4.9, *p* = 0.008). IDH1-mutant and IDH-wild-type patients had a survival of 58.7 (mean) and 31.5 (median) months, respectively. Wang and colleagues 23 demonstrated that IDHwt status was a negative prognostic factor for PFS (HR = 2.6, *p* = 0.001). This finding was confirmed by a 43-patient retrospective study 25 showing the IDHwt status was a negative PFS prognosticator ( *p* = 0.009).

Tang and colleagues 26 identified an intact *1p19q* intact as a significant negative OS prognosticator ( *p* = 0.048) with a survival of 30.0% compared with 77.5% in codeleted patients after 60 months. Codeleted *1p19q* in oligodendrogliomas was a positive PFS prognosticator in two studies. 24 25 A 72-patient molecular analysis showed that *1p19q* codeletion was associated with increased PFS (HR = 0.029, *p* = 0.014). 24

In a retrospective study, 25 Compes and colleagues identified *7p* gain and *10q* loss as a significant positive OS prognosticator ( *p* = 0.016). Improved PFS was also noted in patients with *7p* gain and *10q* loss ( *p* = 0.009), while hypermethylated status and IDHwt astrocytomas as compared with IDH-mutant oligodendrogliomas and diffuse astrocytomas correlated with shorter PFS ( *p* = 0.009). 25

##### Histopathologic

Higher histologic grade was found to be a negative OS prognosticator in four studies. 33 34 35 37 A study by Sughrue et al 37 noted grade III and IV patients had a 2-year survival of 75 and 33%, respectively, after hyperaggressive resection, while 83% of grade II patients survived until the end of follow-up ( *p* < 0.001). Another study by Capizzano et al 35 quote higher neuronal differentiation as a significant positive prognostic factor of OS ( *p* < 0.01). Similarly, Simon and colleagues 33 reported that higher-grade histologic phenotype of glioblastoma was a negative OS and PFS prognosticator ( *p* = 0.004 and 0.017, respectively) when compared with all other histologic types. Conversely, the oligodendroglial phenotype was recognized as a significant positive predictor of OS ( *p* < 0.001) and PFS ( *p* < 0.001) in a retrospective study by Simon and colleagues. 33

In a study by Hameed et al, 27 255 patients with LGGs were divided into two groups based on the MIB-1/K _i_ -67 proliferative indices (PIs): PI >5% and ≤5%. MIB-1/K _i_ -67 PI > 5 was a significant negative predictor of OS (HR = 0.314, *p* = 0.013). Compes et al 25 demonstrated that vimentin positive staining is also a significant negative prognosticator of OS ( *p* = 0.029) and PFS ( *p* = 0.011).

##### Clinical

The presence of permanent deficits was a negative postoperative OS prognosticator in LGGs ( *p* < 0.05) and HGGs ( *p* = 0.005), as reported by Hameed and colleagues 27 LGG patients with no deficit had an OS of 66.89 (mean) compared with 48.00 (median) months in patients with a deficit. In HGG, patients with a transient (resolved by 6 months post-op) or permanent (persisting at 6 months post-op) deficit had an 80 or 0% population survival after 48 months, respectively.

Postoperative KPS 80 to 100 compared with <70 was a positive PFS and OS prognosticator both immediately after the operation ( *p* < 0.001) and after 3 months ( *p* < 0.001) according to Simon and colleagues. 33 Sughrue and colleagues 37 associated KPS < 70 with worse prognosis after hyperaggressive resection ( *p* < 0.001) and increased surgical risk ( *p* = 0.005) in patients with multilobar insular tumors.

#### II. Resectability Predictors

Of the 27 included studies, 17 investigated EOR predictors. 22 24 27 39 40 41 42 43 44 45 46 47 48 49 50 51 52 Sixteen resectability predictors were recorded and are summarized in Table 2 .

**Table 2:** Summary of resectability predictors of INGs detected in the included studies

Prognosticator	Effect on EOR	Study	Study design	Patients ( *n* )
**Preoperative**				
**1. Radiologic**				
Zone I/IV Berger–Sanai tumor	Positive ( *p* = 0.024)	Hameed et al 27	Retrospective	255
Zone I/IV/I + IV Berger–Sanai tumor	Positive ( *p* < 0.01)	Li et al 49	Retrospective	253
Zone II Berger–Sanai tumor	Negative ( *p* = 0.02)	Pitskhelauri et al 44	Retrospective	79
Berger–Sanai giant tumor	Negative ( *p* = 0.024)	Hameed et al 27	Retrospective	255
Intact superior extremity of the central insular sulcus	Positive ( *p* = 0.043)	Kawaguchi et al 22	Retrospective	83
IFOF identification adjacent to tumor	Negative ( *p* = 0.03)	Martino et al 39	Anatomical cadaveric/cohort	10
Insular, opercular, paralimbic, limbic involvement (in order of decreasing EOR)	Negative (no *p* value)	Ozyurt et al 46	Retrospective	40
Encased LSAs	Negative ( *p* < 0.001)	Kawaguchi et al 22	Retrospective	83
Encased LSAs	Negative (no *p* value)	Rao et al 51	Prospective	48
Tumor expansion medially to LSAs	Negative (no *p* value)	Moshel et al 47	Retrospective	25
Encased deep perforators	Negative ( *p* = 0.012)	Rossi et al 50	Retrospective	95
**2. Clinical**				
Seizure control	Positive ( *p* = 0.010)	Rossi et al 50	Retrospective	95
**Intraoperative**				
Transcortical approach (vs. trans-sylvian)	Positive ( *p* < 0.05)	Benet et al 52	Anatomical cadaveric	16
Combined high-field iMRI and functional neuronavigation	Positive ( *p* = 0.031)	Chen et al 41	Retrospective	51
IMRIS 3.0-T iMRI integrated neurosurgical suite	Positive ( *p* = 0.008)	Zhuang et al 42	Retrospective	30
Extensive brain mapping	Positive ( *p* = 0.01)	Rossi et al 50	Retrospective	95
5-ALA fluorescence-guided resection	Positive ( *p* = 0.05)	Barbosa et al 43	Retrospective	28
**Postoperative**				
**1. Histopathologic**				
Higher tumor grade	Positive ( *p* < 0.001)	Hameed et al 27	Retrospective	255
**2. Clinical**				
Seizure control	Positive ( *p* = 0.001)	Rossi et al 50	Retrospective	95

Abbreviations: 5-ALA, 5-aminolevulinic acid; EOR, extent of resection; IFOF, inferior fronto-occipital fasciculus; iMRI, intraoperative magnetic resonance imaging; INGs, insular gliomas; LSAs, lenticulostriate arteries; NR, not reported.

### Preoperative Resectability Predictors

#### Radiologic

Zone I + IV Berger–Sanai tumors were identified as a significant positive resectability predictor ( Fig. 3 ). Hameed and colleagues 27 compared the anterior zone tumors (zones I + IV), posterior zone tumors (zones II + III), superior zone tumors (zones I + II), and inferior zone tumors (zones III + IV). The anterior zone tumors had the greatest EOR ( *p* = 0.024). Similarly, Li and colleagues 49 reported anterior tumors (Berger–Sanai zones I, IV, and I + IV) to have a significantly higher GTR than posterior type, anteroposterior type, and giant-type tumors ( *p* < 0.01) in a retrospective study of 253 INGs operated using a transcortical (TC) approach. However, a retrospective study on ING recurrence by Morshed and colleagues 40 found that EOR during reoperation was not impacted by the Berger–Sanai zone.

The Berger–Sanai zone II tumor location was a negative resectability predictor identified by Pitskhelauri and colleagues. 44 Fewer zone II tumors achieved EOR ≥ 90% and they were most strongly associated with residual tumor in 40.0% of cases ( *p* = 0.02).

Intact superior extremity of the central insular sulcus is positively associated with GTR in a retrospective study on 83 patients ( *p* = 0.043). 22 GTR was achieved in 20.7% of patients with and 57.4% of patients without tumor extension to this location ( *p* = 0.001).

In contrast, Berger–Sanai giant tumor was identified as a negative resectability predictor ( *p* = 0.024). The EOR was the lowest for giant tumors (median = 93.6%; interquartile range [IQR] = 83.5–100%) when compared with ASPI zone tumors. 27

Preoperative inferior fronto-occipital fasciculus (IFOF) identification through diffusion tensor imaging (DTI) was highlighted as a negative resectability predictor ( *p* = 0.03) in an anatomical cadaveric/cohort study by Martino and colleagues 39 and was also associated with EOR < 80%. EOR > 80% was observed in 71.4 and 87.5% of cases with and without preoperative IFOF identification, respectively.

Ozyurt and colleagues 46 introduced an MRI-based topological classification system and examined its effect on EOR. A comparable EOR was achieved in purely insular tumors and tumors extending to the opercula, with total resection in 71 and 75%, respectively. This decreased to 67% in tumors extending to the paralimbic structures and 30% in tumors extending to limbic structures. No analysis of statistical significance was performed.

The most commonly reported resectability predictor was nonencased LSAs. 22 47 51 53 Kawaguchi and colleagues 22 found that LSAs, visualized by MR microangiography, were significantly associated with GTR ( *p* < 0.001; odds ratio [OR]: 35.5; 95%CI: 6.02–209.2). Rao and colleagues 51 determined that the percentage of patients with GTR was higher in the LSA-pushed group (GTR = 42.8%) than in the LSA-encased (GTR = 5%). Moshel and colleagues 47 used tumor position relative to the LSAs as a radiologic predictor. GTR or near-total resection was achieved in 84% of patients with tumors lateral to the LSAs compared with 54% of medially expanding tumors.

In contrast, Rossi and colleagues 50 reported encasement of other deep perforators, as a significant negative resectability predictor compared with their medial displacement with GTR achieved in 56.2 and 82.5% of these patient groups, respectively ( *p* = 0.012).

Tumor lateralization in the dominant or nondominant hemisphere was not a significant EOR predictor according to Morshed and colleagues 40 ( *p* = 0.56) and Eseonu and colleagues 24 ( *p* = 0.492).

##### Clinical

Rossi and colleagues 50 identified preoperative seizure control while on antiepileptic medications as a positive resectability predictor in a study of 95 giant INGs. GTR was achieved in 84.6 and 60.5% of patients with adequate and poor seizure control, respectively ( *p* = 0.01).

### Intraoperative Resectability Predictors

A study on 16 cadaveric specimens by Benet and colleagues 52 demonstrated that the TC approach was associated with higher EOR compared with the trans-sylvian (TS) approach. However, a retrospective study on 100 patients showed no significant difference in EOR obtained through the two different surgical approaches. 48

The use of intraoperative adjuncts such as intraoperative MRI (iMRI) and neuronavigation was identified as a positive resectability predictor. 41 42

Both intraoperative technologies were associated with higher EOR in two studies. 41 42 In a cohort study 41 on insular HGGs, the median EOR after 3 months in the iMRI-assisted group was 96% compared with 84% in the conventional neuronavigation group ( *p* = 0.031). Furthermore, the use of 3.0-T iMRI integrated neurosurgical suite by Zhuang and colleagues 42 revealed residual tumor in 26 cases and led to further resection in 9 cases. The percentage of GTR and near-total resection increased from 53 to 77% ( *p* = 0.016).

Rossi and colleagues 50 showed that significantly more patients undergoing extensive brain mapping incorporating nonverbal cognitive, haptic, and visual cues achieved GTR compared with patients undergoing brain mapping limited to motor function and language (81.8 vs. 46.7%; *p* = 0.01).

Additionally, Barbosa and colleagues 43 associated 5-aminolevulinic acid (5-ALA) fluorescence-guided resection with higher EOR. EOR ≥ 90% was achieved in 67 and 24% of resections with and without 5-ALA use, respectively ( *p* = 0.05).

### Postoperative Factors Associated with EOR

#### Histopathologic

Higher tumor grade was identified as a significant positive resectability predictor. In a cohort study by Hameed and colleagues 27 on 255 patients, EOR was higher for HGGs compared with LGGs (median = 98.9 vs. 95.2%, *p* < 0.001).

##### Clinical

Rossi and colleagues 50 reported that postoperative seizure control is associated with increased EOR in a study including patients treated with anti-epileptic medications postoperatively when required. Seizure control status included patients belonging to class I of the Engel Surgical Outcome Scale. Seizure control was achieved in 98.5% of patients undergoing GTR compared with only 76% of patients undergoing STR ( *p* = 0.001).

##### Molecular

Wu and colleagues 45 investigated the effect of 1p/19q codeletion on the EOR. Greater average EOR was observed in the 1p/19q codeletion group (90.1 ± 6.8%) compared with the 1p and/or 19q intact group (70.3 ± 26.9%). However, the results did not reach significance ( *p* = 0.07).

## Discussion

To the best of our knowledge, this is the first systematic review focusing on prognostic factors or resectability predictors unique to INGs. We identified 8 OS prognosticators, 9 PFS prognosticators, and 12 resectability predictors.

Putaminal involvement was a significant negative OS/PFS prognosticator. 23 Tumors involving the putamen were larger, more often IDHwt, and less likely to be completely resected. 23 BG involvement, including globus pallidus, is also a significant negative OS prognosticator. 37 Since the putamen has a strong structure juxtaposed to the insular cortex, involvement is indicative of an infiltrative tumor.

Berger–Sanai giant tumor was identified as a significant negative OS and PFS predictor by Hameed and colleagues, 27 attributed to the larger TC window required for resection.

The prognostic significance of paralimbic involvement was debated in different studies, possibly due to the genetic heterogeneity of paralimbic gliomas. These are more often of the IDHwt phenotype than purely INGs, which are more likely to have IDH1 mutations, associated with favorable outcome, and smaller tumor volume ( *p* < 0.007). 26 The IDHwt phenotype, implicated with worse prognosis, is more common in paralimbic INGs leading to their association with shorter OS and PFS than purely insular tumors, as in the study by Tang and colleagues. 26 However, this review concludes that paralimbic gliomas *should not* be considered as a single entity. Paralimbic IDH-mutant tumors share the same proliferative growth pattern and microRNA as purely INGs that also frequently carry the IDH1-mutant, 26 suggesting that the genetic makeup of tumors is a more sensitive prognosticator than topology.

Nevertheless, tumor location often relates to the origin of tumor precursor cells and subsequently to their genetic makeup. 54 Evidence suggests LGG precursor cells bearing IDH mutation may be region specific. Frontal and INGs, with the highest IDH-mutant rates among gliomas, are both in close proximity to the subventricular zone (SVZ) of neural progenitor cells. 55 56 The SVZ is, in turn, adjacent to the area around the rostral extension of the lateral ventricle, reported as the cellular origin of IDH-mutant glioblastomas. 55 This may justify the high distribution of IDH-mutant gliomas to the insula, frontal lobe, or both, and the higher occurrence of frontoinsular growth pattern in IDH-mutant than in IDH-wild-type paralimbic gliomas. This is likely why Simon and colleagues reported the best prognosis to be associated with Yaşargil type 5A (frontoinsular) and B (frontoinsulotemporal) tumors 33 and concluded that paralimbic involvement is a positive prognosticator. Alternatively, the favorable prognosis could be due to ease of obtaining surgical window using the frontal approach to tackle frontally extending tumors. 33

Examining the resectability predictors, this review recognized the anterior insular tumor location (zone I, IV, and I + IV Berger–Sanai tumors) as a significant positive resectability predictor, 27 49 while the posterior Berger–Sanai zone II and giant tumors were significant negative resectability predictors. 27 44 Posterior and posterosuperior tumors have a lower EOR due to their proximity to the corticospinal tracts and LSAs. This effect disappeared on reoperation when operative corridors were created during the first resection. 40

Intact superior extremity of the central insular sulcus is also a significant positive resectability predictor. 22 This landmark corresponds to Berger–Sanai zone II, the long insular perforator arteries, and the arcuate fasciculus. It is also adjacent to the rolandic cortex, inferior parietal language sites, and posterior limb of the internal capsule. Therefore, tumors with that location relate to lower resectability and higher occurrence of postoperative deficits such as hemiparesis, aphasia, and hemispatial neglect.

Interestingly, IFOF identification in the proximity or within the tumor preoperatively was a negative resectability predictor. 39 DTI reconstruction of the tract often fails in infiltrative tumors and direct electrical stimulation does not identify eloquent subcortical areas in the proximity, likely due to complete disruption of the tract. Lack of eloquent areas allows the deep functional margin of resection to extend to the striatum and increase the EOR. In contrast, nondiffusive tumor growth results in medial IFOF displacement and its successful identification and use as deep functional limit of the resection. Residual tumor below that margin remains, thus decreasing EOR. 39 This may also be the consequence of the reluctance of neurosurgeons to proceed below the IFOF margin, to avoid damaging the optic radiation and LSAs. 57

Additionally, nonencased LSAs emerged as the most commonly reported resectability predictor in the literature. 22 47 51 53 LSA localization can be achieved utilizing three-dimensional time-of-flight MRI combined with a technique to superimpose LSA position on T2-weighted imaging delineating tumor margins. 22 47 51 53 This may involve use of T2-gradient echo sequences such as three-dimensional constructive interference in steady state 51 and transformation of data to cine images for review of the tumor–LSA interface. 22 53 Hence, LSAs can be detected as high-intensity spots in the white matter. Nonencased LSAs will be pushed, facilitating tumor resection and increasing EOR. 47 51 The percentage LSA shift can be calculated as the distance between the maximally deviated LSA and the sylvian fissure divided by the distance of the LSA origin from the sylvian fissure. 47

An anatomical cadaveric study 52 on 16 specimens found the TC approach to be a positive resectability predictor with better exposure for zone I, III, and IV insular tumors and greater surgical freedom in zones I, II, and III compared with the TS. On the contrary, Przybylowski and colleagues found no effect of the different surgical approaches on the EOR. 48 This may be due to higher average tumor volumes than those in the study by Benet and colleagues 52 influencing the choice of surgical technique toward the TC approach for high-volume cases. 48

A well-documented positive intraoperative prognostic factor of OS and PFS discussed in five studies is higher EOR, with EOR ≥90% most consistently quoted as a significant positive prognosticator. Intraoperative MRI and functional neuronavigation are significant positive resectability predictors that identify residual tumor, often prompting further resection. 41 42 Combination of iMRI and functional neuronavigation is more effective than functional neuronavigation alone in increasing EOR and reducing residual tumor volume, especially where they are too small to be otherwise detected or in infiltrative tumors where the tumor borders are not easily identifiable. 41 42 Additional use of iMRI can update neuronavigation to account for intraoperative brain shift. It reduces morbidity by identifying functional speech areas, the arcuate fasciculus, and, when combined with DTI, it facilitates the preservation of the pyramidal tract. 41 42

Presentation with epileptic seizures was both a positive OS and PFS prognosticator when compared with presentation with any other symptom or without seizures. While traditionally regarded as an indicator of tumor progression, 58 epileptic seizures have been linked to IDH-mutant gliomas with better prognosis. 59 60 61 62 Mutant IDH1 increases d-2-hydroxyglutarate (D2HG), a glutamate mimetic contributing to increased neuronal excitation and seizures. 62 Moreover, according to Wang and colleagues, 23 the larger putaminal-invading tumors and glioblastomas were less likely to present with seizures than purely INGs. This could be explained by higher frequency of IDH mutation in purely insular tumors. The emerging relationship between noninvasively assessed clinical symptoms such as seizure occurrence and molecular tumor characteristics should be further explored.

Interestingly, preoperative seizure control was a positive resectability predictor and postoperative seizure control defined as Engel surgical class I was associated with increased EOR. Still and colleagues 63 also previously reported that an EOR ≥ 91% and a residual tumor volume ≤19 cm ^3^ are associated with better postoperative seizure control. They postulated that epileptic activity arises from cortical residual tumor rather than deep residual tumor seated in white matter tracts. 63 Hence, preoperative poor seizure control may indicate cortical eloquent tumor location, which will not be completely resected. Similarly, poor postoperative seizure control reflects residual tumor in eloquent areas identified via brain mapping that was not resected to avoid neurologic deficit and functional status deterioration.

Several clinical pre- and postoperative prognosticators related to the functional status of patients were identified. Permanent deficits postoperatively, as opposed to transient deficits, are a negative OS prognosticator. 27 This factor is highly associated with topology, since tumor location predicts the probability and type of postoperative deficit. According to Hameed and colleagues, 27 the majority (84.21%) of patients with postoperative language deficits had either a giant or a posterior insular tumor, while the majority (86.96%) of patients with postoperative motor deficits had either a giant or an anterior insular tumor. Motor deficits have been especially associated with decreased OS. 64 Furthermore, patients with giant tumors were more likely to experience postoperative deficits (relative risk: 1.58; *p* = 0.038). Furthermore, preoperative KPS ≥ 90 and postoperative KPS ≥ 80 both immediately and after 3 months were positively correlated with OS and PFS. 23 33 36 As discussed, the prognostic value of KPS relates to detecting the effects of pre- and postoperative deficits according to the location and extent of tumor infiltration.

Six studies examined the effect of histologic markers on PFS/OS. Vimentin positive staining, 25 higher tumor grade, 34 35 37 and glioblastoma phenotype 33 were found to be significant negative prognostic factors of OS and PFS. The oligodendroglioma (compared with astrocytoma) phenotype 33 was a significant positive prognosticator of OS and PFS. MIB-1/K _i_ -67 PI < 5 27 and higher neuronal differentiation 35 are significant positive prognosticators of OS. No histologic prognosticator is specific to the insula and most, including vimentin staining and MIB-1/K _i_ -67 PI, owe their prognostic effect to their association with tumor grade.

Anatomical, developmental, molecular, histopathologic, and clinical factors intertwine in INGs to affect their prognosis and resectability. Yaşargil's anatomical and developmental classification of limbic and paralimbic tumors 65 showed limited prognostic value, perhaps due to omission of biological tumor characteristics. The Berger–Sanai 3 classification is most practical for surgical resection planning and the prognostic putaminal 23 classification uses invasiveness of anatomical structures—possibly related to molecular characteristics. Integration of the three classifications, combined with topology-associated molecular tumor patterns and clinical patient characteristics to form a unified ING classification system, could simplify practice and improve clinical decision-making.

## Limitations

This analysis is limited by the lack of high-quality evidence on ING prognosticators and resectability predictors. Most studies relied on retrospective analysis with inconsistent follow-up times and no multi-institutional cohorts were included. OS, PFS, and prognostic factors affecting them were identified on patients undergoing surgical resection. Surgical randomization was not achieved due to the lack of a clinical equipoise, making surgical treatment a confounding factor when prognosticators are applied to presenting patients. Three reports included were rated as moderate to high RoB due to insufficient adjustment for cofounders and inadequate statistical analyses. Furthermore, a meta-analysis was not possible due to the heterogeneity of the variables included in the HRs across different studies. Standardization of these variables could facilitate data collation in future studies.

## Conclusions

Negative prognosticators reported in ≥2 studies included putaminal or paralimbic involvement and higher tumor grade, while seizures at presentation, IDH mutation, increased EOR, and higher KPS score preoperatively and at 3 months postoperation were positive prognosticators. Resectability predictors reported in ≥2 studies included zone I/IV tumor, encased LSAs, and use of intraoperative imaging. A large prospective trial to evaluate the significance of the identified OS, PFS, and resectability predictors is needed to devise a grading system for INGs and assist in personalized clinical management.
